# Development and validation of fat-corrected virtual MR elastography to assess fibrosis stage in metabolic dysfunction-associated steatotic liver disease

**DOI:** 10.1186/s13244-026-02287-4

**Published:** 2026-04-18

**Authors:** Jie Yuan, Xinxin Xu, Fuhua Yan, Zhiwei Qin, Huamei Yan, Wenjie Yang, Huimin Lin

**Affiliations:** 1 Department of Radiology, Ruijin Hospital, Shanghai Jiao Tong University School of Medicine, Shanghai, China; 2 Department of Radiology, Shuguang Hospital Affiliated to Shanghai University of Traditional Chinese Medicine, Shanghai, China; 3 College of Health Science and Technology, Shanghai Jiao Tong University School of Medicine, Shanghai, China; 4 MR Research Collaboration, Shanghai United Imaging Healthcare Co., Ltd., Shanghai, China; 5 Clinical Research Center, Shuguang Hospital Affiliated to Shanghai University of Traditional Chinese Medicine, Shanghai, China

**Keywords:** Fat correction, Virtual magnetic resonance elastography, Metabolic dysfunction-associated steatotic liver disease, Diffusion-weighted imaging

## Abstract

**Objectives:**

This study aimed to develop and validate a fat-corrected virtual magnetic resonance elastography (FC-vMRE) framework based on diffusion-weighted imaging (DWI) to assess liver fibrosis in patients with metabolic dysfunction-associated steatotic liver disease (MASLD).

**Materials and methods:**

A total of 463 MASLD patients underwent multi-*b*-values (0–1500 s/mm²) DWI acquisition, proton density fat fraction (PDFF), and conventional magnetic resonance elastography (MRE). Apparent diffusion coefficient (ADC) parameters were calculated with or without fat correction (based on PDFF). Using the training cohort (*n* = 361), ADC-to-MRE formulas were derived to compute virtual MRE (vMRE) and FC-vMRE. In the validation cohort (*n* = 102, with biopsy), the diagnostic performance of vMRE, FC-vMRE, and MRE for fibrosis staging was compared.

**Results:**

ADC_200-1200_ demonstrated the strongest correlation with MRE values (*R* = −0.706, *p* < 0.001), yielding the formula: FC-vMRE = 6.50 − 3.13 × ADC*w*. Compared with vMRE, FC-vMRE showed superior agreement with MRE (bias: −0.001 kPa vs 0.469 kPa; intraclass correlation coefficient: 0.666 vs 0.381). In staging performance, FC‑vMRE significantly outperformed vMRE across all fibrosis stages. FC-vMRE showed promising diagnostic performance to MRE in detecting ≥ F2 fibrosis (AUC: 0.761 vs 0.848, *p* = 0.053), ≥ F3 fibrosis (AUC: 0.756 vs 0.818, *p* = 0.066), and cirrhosis (AUC: 0.838 vs 0.914, *p* = 0.065).

**Conclusion:**

FC-vMRE provides a clinically promising alternative requiring only standard MRI equipment, effectively correcting for fat-related confounding in MASLD, and demonstrating encouraging diagnostic concordance with MRE and histology. This approach shows potential for broader clinical implementation.

**Critical relevance statement:**

Fat-corrected virtual MRE using diffusion-weighted imaging provides promising fibrosis staging accuracy to conventional magnetic resonance elastography in metabolic dysfunction-associated steatotic liver disease patients without requiring specialized hardware.

**Key Points:**

- Fat infiltration confounds diffusion measurements in metabolic steatotic liver disease.
- Fat-corrected virtual MRE showed superior agreement with standard MRE.
- Hardware-free fibrosis assessment enables accessible liver stiffness measurement.

**Graphical Abstract:**

**Figure:**
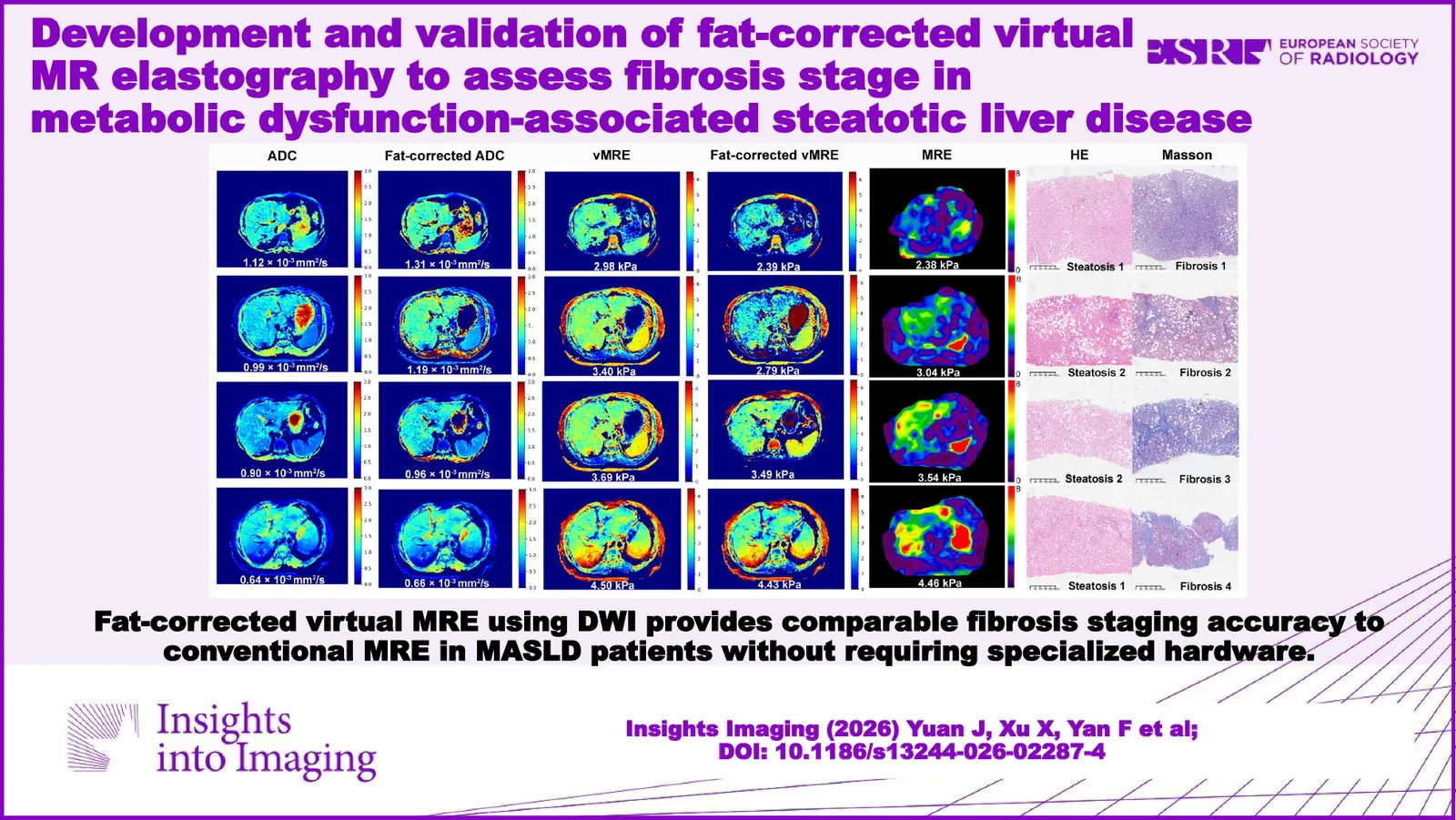

## Introduction

Metabolic dysfunction-associated steatotic liver disease (MASLD) affects approximately 25% of the global population, posing a substantial healthcare burden through its strong association with metabolic syndrome [1, 2]. The MASLD spectrum progresses from simple steatosis to metabolic dysfunction-associated steatohepatitis (MASH), cirrhosis, and hepatocellular carcinoma. The presence of fibrosis is the most important prognostic factor in MASLD and is correlated with liver-related outcomes and mortality, necessitating accurate fibrosis assessment for prognostic stratification and therapeutic guidance [3].

While liver biopsy remains the gold standard for diagnosing and staging liver fibrosis, its invasive nature, potential complications, and sampling variability have led to the development of noninvasive diagnostic methods [4]. Magnetic resonance elastography (MRE) has emerged as a highly accurate technique for this purpose, demonstrating superior diagnostic performance compared to ultrasound-based elastography [5, 6]. MRE quantifies liver stiffness by visualizing propagating mechanical waves within the tissue, providing a robust biomarker strongly correlated with fibrosis stage. However, widespread MRE adoption faces significant challenges due to specialized hardware requirements, increased examination time, technical complexity, and higher costs.

Consequently, there is considerable interest in leveraging standard MRI sequences for fibrosis assessment. Diffusion-weighted imaging (DWI), a routine component of liver MRI protocols, measures the random motion of water molecules. The apparent diffusion coefficient (ADC) derived from DWI reflects tissue microstructure, including influences from cellularity, extracellular space, and fibrosis [7]. This has led to the development of virtual MRE (vMRE) concepts, which utilize ADC values to estimate liver stiffness non-invasively, offering the potential for hardware-free assessment using widely available sequences. The vMRE methodology is based on the principle that restricted water diffusion in fibrotic tissue, as measured by DWI at high *b*-values, correlates strongly with tissue stiffness. Specifically, DWI acquisitions are performed using multiple *b*-values, and pixel-by-pixel fitting generates ADC maps. A shifted ADC calculated from high *b*-values is then converted to shear modulus through empirically derived calibration equations established against standard MRE measurements [8]. However, translating vMRE to MASLD presents distinct challenges. Unlike other liver diseases, MASLD presents unique challenges, characterized by a distinct triad of pathological changes—steatosis, inflammation, and fibrosis—which occur in varying degrees and combinations throughout disease progression [9]. Appropriate *b*-values selection for DWI acquisition may differ between MASLD and other chronic liver diseases. Crucially, fat confounds the observed ADC in patients with hepatic steatosis, a hallmark feature of MASLD [10]. This confounding effect can lead to inaccurate DWI interpretations and impact vMRE measurements. Recent advances in quantitative fat fraction mapping using proton density fat fraction (PDFF) have enabled the development of fat-correction algorithms that can derive fat-corrected ADC values (ADC*w*) from conventional ADC measurements, potentially improving the accuracy of diffusion-based liver fibrosis assessment in MASLD patients [11]. This study aims to develop and validate a novel fat-corrected vMRE (FC-vMRE) framework using clinical-grade DWI acquisitions specifically optimized *b*-values for MASLD. By establishing diagnostic concordance with MRE and histological reference standards, we seek to provide a clinically viable, hardware-free alternative for comprehensive MASLD evaluation.

## Materials and methods

### Study cohort

This prospective study received approval from the Institutional Review Board of Shuguang Hospital affiliated with Shanghai University of Traditional Chinese Medicine (2023-1249-16-01), and all participants provided written informed consent. Patients were preselected from the hepatology and metabolic disease clinics based on known or clinically suspected hepatic steatosis, identified through previous imaging, abnormal liver enzymes, or metabolic risk profiles. From this preselected population, the study consecutively enrolled adults (≥ 18 years) between January 2022 and June 2024 who underwent liver MRI assessment and met the following criteria: (1) MRI-proton density fat fraction (MRI-PDFF) confirmed hepatic steatosis (≥ 5% liver fat content); and (2) at least one metabolic risk factor for MASLD: BMI > 23 kg/m²; fasting blood glucose ≥ 5.6 mmol/L; triglycerides ≥ 1.7 mmol/L or on lipid-lowering treatment; high-density lipoprotein-cholesterol: ≤ 1.0 mmol/L for males, ≤ 1.3 mmol/L for females, or on lipid-lowering treatment; blood pressure > 130/85 mmHg or on hypertension treatment [3]. Exclusion criteria included significant alcohol consumption (> 30 g/day for men, > 20 g/day for women) and other chronic liver diseases. Between January 2022 and June 2024, participants were divided into a training cohort and a validation cohort. The training cohort comprised patients without liver biopsy, from whom the fat-corrected virtual MRE model was developed using noninvasive imaging parameters. The validation cohort comprised patients who underwent liver biopsy for clinical indications, providing histopathological reference standards for model validation.

### MRI acquisition

All imaging was performed on a 3.0-T MRI system (uMR 790, United Imaging Healthcare) with a 32-channel flexible body coil. All subjects fasted for a minimum of 6 h before MRI scanning. Three sequences were utilized: MRE, DWI, and PDFF. For the MRE, a 60 Hz controller and driver were employed. The sequence parameters were as follows: repetition time (TR)/echo time (TE) = 1000.2/44.6 msec; field of view (FOV) = 420 × 420 mm^2^; matrix = 96 × 96; slice thickness = 8 mm; acceleration factor = 2; acquisition time = 10 s. Shear stiffness maps were automatically generated from the acquired wave images. DWI utilized a respiratory-navigated, fat-suppressed echo-planar imaging (EPI) technique. Parameters: TR/TE = 3000/69.9 msec; slice thickness = 7 mm; FOV = 380 × 280 mm^2^; matrix = 144 × 144; *b*-values of 0, 50, 100, 150, 200, 800, 1000, 1200 and 1500 s/mm². Averages: one for *b* ≤ 200 s/mm^2^, three for *b* ≥ 800 s/mm^2^; acquisition time = 7 min and 57 s. For PDFF, 3D Fat Analysis & Calculation Technique (FACT) sequence used chemical shift encoding. Parameters: TR/TE = 12.06/1.71, 3.22, 4.73, 6.24, 7.75, 9.26 msec; flip angle = 3°; FOV = 420 × 420 mm^2^; matrix = 240 × 240; slice thickness = 10 mm; bandwidth = 900 Hz; acceleration factor = 2; acquisition time = 15 s.

### MRI analysis

Original MR images were transferred to the specific workstation (uWS, United Imaging Healthcare) for data analysis. Quantitative assessments included MRE stiffness and PDFF measurements, and ADC calculations from DWI.

**Fig. 1:**
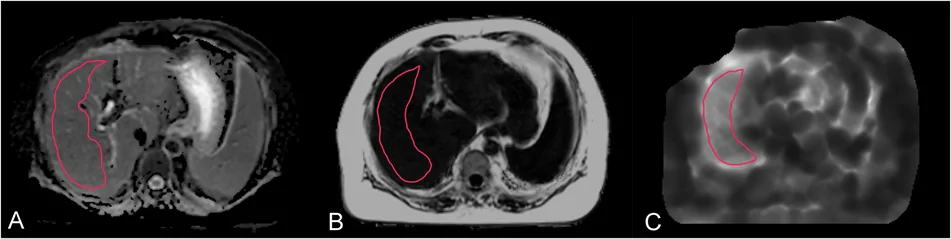
Representative region of interest (ROI) placement on multiparametric MRI maps in a patient with metabolic dysfunction-associated steatotic liver disease (MASLD). **A** Apparent diffusion coefficient (ADC) map, (**B**) proton density fat fraction (PDFF) map, and (**C**) magnetic resonance elastography (MRE) elastogram

Liver stiffness was assessed by manually delineating polygonal regions of interest (ROIs) on the largest measurable hepatic sections across four elastograms [12]. Four ROIs were placed on the largest cross-sectional hepatic slices while carefully avoiding major vessels, bile ducts, and organ boundaries. Each ROI measured greater than 2.5 cm² to ensure adequate sampling while maintaining distance from potential confounding structures. The mean stiffness value was calculated from these four measurements. Two radiologists, a senior with 12 years and a junior with 5 years of abdominal MRI experience, collaboratively determined the ROIs through consensus reading, discussing any uncertain areas until agreement was reached. The MRE, PDFF and DWI ROIs were aligned at the same level where possible, as shown in Fig. 1.

ADC values were computed using nine different *b*-value combinations (low *b*-values: 0, 50, 100, 150, 200 s/mm²; high *b*-values: 800, 1000, 1200, 1500 s/mm²) according to the mono-exponential model:

1 $${ADC}=\frac{{{\mathrm{ln}}}\left(\,S1/S2\,\right)}{b2-b1}$$

Where *S*1 and *S*2 represent signal intensities at low (*b*1) and high (*b*2) *b*-values, respectively. To account for confounding fat signal contributions, all ADC values underwent correction using the algorithm described by Hansmann et al [11].

2 $$S\left(b\right)=A\left(\left(1-\eta \right){e}^{-\frac{{TE}}{{T2}_{w}}}{e}^{-b{{ADC}}_{w}}+\alpha \eta {e}^{-\frac{{TE}}{{T2}_{f}}}{e}^{-b{{ADC}}_{f}}\right)$$

where *A* is the local proton density, *η* is the PDFF, *α* is the fraction of residual fat signal that is unsuppressed or has been excited, *TE* is the echo time, *T2*_*w*_ and *T2*_*f*_ are the T2 relaxation times of the water and fat, respectively, and *ADC*_*w*_ and *ADC*_*f*_ are the true ADCs of water and fat, respectively. According to Hansmann et al, *T2*_*w*_ = 23 msec and *T2*_*f*_ = 62 msec, *ADC*_*f*_ = 0 sec/mm^2^, α = 0.087. Thus, the fat-corrected ADC (*ADC*_*w*_) can be derived by the above equation.

To assess measurement reproducibility, inter-reader reproducibility was assessed in a randomly selected 20% subset (*n* = 72) of the training cohort using intraclass correlation coefficients (ICC). Detailed ICC values and results are provided in the Supplementary Materials.

### Development of FC-vMRE model

The development of FC-vMRE was performed in the training cohort to establish ADC-to-MRE conversion formulas. This process involved the following steps: First, correlation analysis was conducted between all twenty ADC*w* parameters and actual MRE-measured liver stiffness values using correlation coefficients. This analysis identified which ADC*w* parameter exhibited the strongest relationship with liver stiffness. Second, linear regression modeling was performed to derive the mathematical relationship between the optimal ADC parameter and MRE values. A regression model was established, using ADC*w* derived from Equation [2] to generate the FC-vMRE formula. The regression equation took the general form:

3 $${{{\rm{FC}}}}-{{{\rm{vMRE}}}}={{{\rm{\alpha }}}}+{{{\rm{\beta }}}}\times {{{\rm{ADC}}}}w$$

where α represents the intercept and β represents the slope coefficient.

### Liver histology

Patients were diagnosed with MASLD according to the Brunt criteria, and liver biopsy samples were scored using the NASH-CRN histological scoring system [13]. Histopathological assessment was performed independently by two experienced histopathologists (with > 10 years of experience in liver pathology), who were blinded to all clinical data and imaging results including MRI and MRE findings. In cases of discordance, a consensus was reached through joint re-evaluation. The median interval between MRI examination and liver biopsy was 8 days (range: 0–89 days; interquartile range: 2–12 days).

### Statistical analysis

Statistical analyses were performed using R version 4.3.1. Continuous variables were expressed as mean ± standard deviation for normally distributed data or median for non-normally distributed data, determined by the Shapiro–Wilk test. Pearson or Spearman correlation coefficients evaluated relationships between FC-vMRE and MRE values. Bootstrap resampling (1000 iterations) assessed correlation coefficient stability across ADC*w* parameter combinations using 95% confidence intervals. Linear regression derived the FC-vMRE formula using optimal ADCw parameters as independent variables and MRE as the dependent variable. Agreement between MRE, vMRE, and FC-vMRE was quantified using Pearson’s correlation, Bland–Altman analysis with 95% limits of agreement, and intraclass correlation coefficients (ICC). For fibrosis staging in the validation cohort, 10-fold stratified cross-validation was performed to determine optimal cutoff values and calculate threshold-based diagnostic metrics (sensitivity, specificity, PPV, NPV, and accuracy), which were reported as mean ± standard deviation across folds to avoid overfitting bias. In each fold, optimal cutoff values were determined in the training set by maximizing Youden’s index, then applied to the independent test set for performance evaluation. Receiver operating characteristic (ROC) curves and areas under ROC curves (AUC) with 95% confidence intervals were calculated using the full validation cohort dataset, and AUC values were compared using DeLong’s test. Calibration analyses were conducted to evaluate agreement between predicted probabilities and observed outcomes across all fibrosis thresholds in the Supplementary Materials. Statistical significance was set at *p* < 0.05.

## Results

### Study population demographics and characteristics

**Fig. 2:**
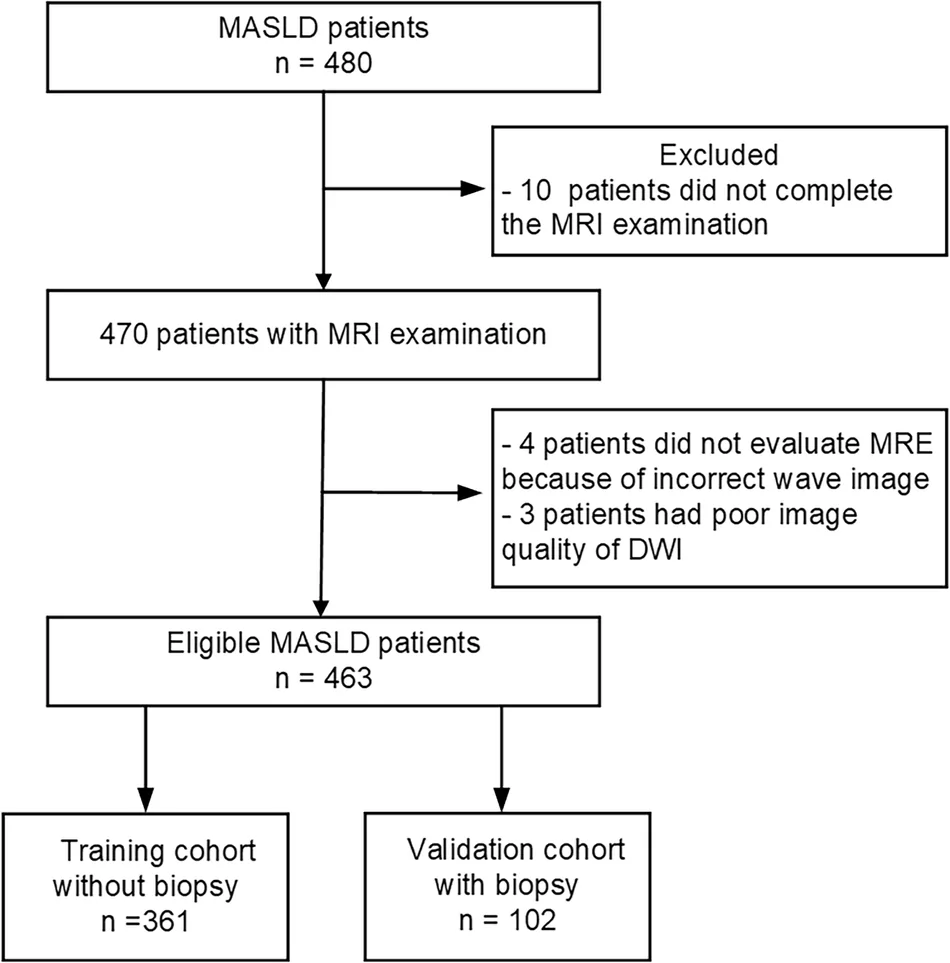
Flowchart

A total of 463 patients were included in this study, comprising 361 patients in the training cohort and 102 patients in the validation cohort, as shown in Fig. 2. In the validation cohort, fibrosis stages were distributed as follows: F0 (*n* = 2, 2.0%), F1 (*n* = 25, 24.5%), F2 (*n* = 38, 37.3%), F3 (*n* = 24, 23.5%), and F4 (*n* = 13, 12.7%). The training cohort had a median age of 48.00 years (22.00–82.00 years) with 60.9% male patients, while the validation cohort had a median age of 49 years (18.0–77.0 years) and a male proportion of 47.5%. Median MRE values were higher in the validation cohort (3.64 kPa) compared to the training cohort (2.96 kPa). Table 1 presents the detailed patient demographics and histological findings of the validation group.

**Table 1:** Patient characteristics

	Training cohort (*n* = 361)	Validation cohort (*n* = 102)
Demographics		
Age, years (median, IQR)	48.00 (22.00–82.00)	49.00 (18.00–77.00)
Gender, male, *n* (%)	60.9	47.5
BMI (kg/m^2^)-median (IQR)	26.00 (24.20–27.75)	25.39 (23.44–28.73)
Blood-median (IQR)		
ALT (IU/L)	45.00 (28.00–72.00)	68.00 (39.75–112.75)
AST (IU/L)	35.00 (25.26–52.75)	58.00 (34.00–91.00)
Platelets count (× 10^9^/L)	216.00 (182.00–255.00)	192.00 (155.00–233.00)
Triglyceride (mg/dL)	1.81 (1.23–2.48)	1.63 (1.27–2.16)
Total cholesterol (mg/dL)	5.24 (4.45–5.73)	5.06 (4.39–5.55)
HDL cholesterol (mg/dL)	1.12 (0.97–1.26)	1.10 (0.96–1.32)
LDL cholesterol (mg/dL)	3.07 (2.58–3.63)	2.90 (0.96–3.39)
FBG (mmol/L)	5.30 (5.00–5.90)	5.60 (5.00–6.60)
MRI-median (IQR)		
PDFF (%)	12.20 (7.10–19.15)	12.70 (6.72–18.37)
MRE (kPa)	2.96 (1.61–5.50)	3.64 (2.27–6.03)

*BMI* body mass index, *IQR* interquartile ratio, *ALT* alanine aminotransferase, *AST* aspartate aminotransferase, *HDL* high-density lipoprotein, *LDL* low-density lipoprotein, *FBG* fasting blood glucose, *PDFF* proton density fat fraction, *MRE* magnetic resonance elastography

### Correlation analysis between ADC parameters and MRE values

**Fig. 3:**
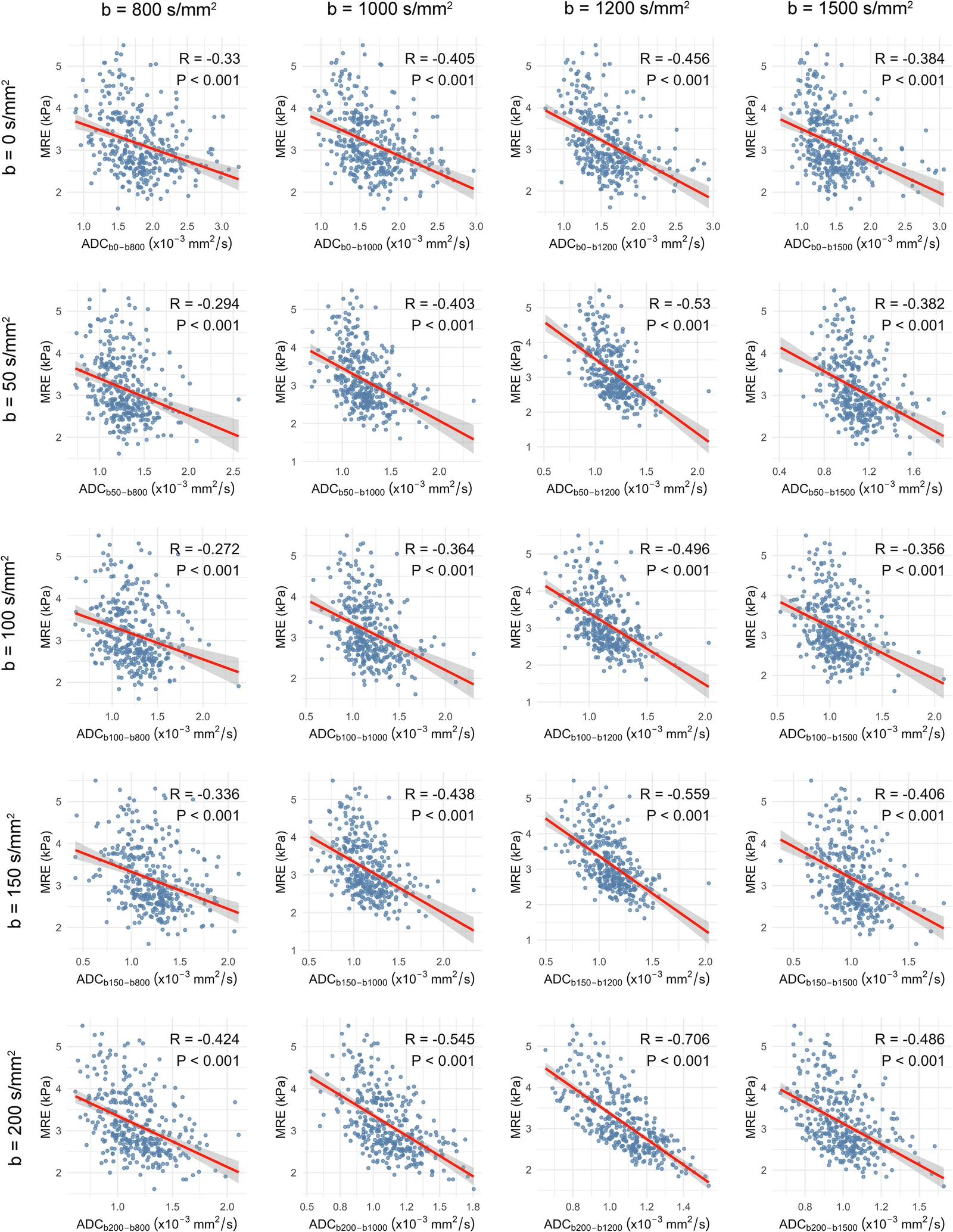
Correlation analysis between various ADC_*w*_ parameters and MRE measurements. Scatter plots illustrating the relationships between 20 distinct ADC_*w*_ parameters and corresponding MRE values. Each subplot represents a specific ADC_*w*_ parameter combination, plotted against MRE measurements. The *x*-axis denotes the ADC_*w*_ value (× 10⁻³ mm²/s) for each parameter, while the *y*-axis represents the MRE-derived liver stiffness (kPa)

In the training cohort, all fat-corrected ADC_*w*_ parameter combinations exhibited significant negative correlations with MRE values (all *p* < 0.001). Figure 3 illustrates these inverse relationships through scatter plots, revealing consistent distribution patterns. The strongest correlation was observed for fat-corrected ADC_200-1200_ (R = −0.706, *p* < 0.001), followed by ADC_150-1200_ (*R* = −0.559, *p* < 0.001) and ADC_200-1000_ (R = −0.545, *p* < 0.001). Bootstrap analysis with 1000 replicates confirmed the consistent superiority of ADC_200-1200_ over alternative parameter combinations. All correlation differences (Δr) ranged from −0.146 to −0.209, with 95% CIs excluding zero (*p* < 0.05). Given its superior correlation strength, fat-corrected ADC_200-1200_ was selected as the optimal parameter for FC-vMRE calculation. The derived FC-vMRE formula was:

4 $${{{\rm{FC}}}}-{{{\rm{v}}}}{{{\rm{MRE}}}}=6.50-3.13\times {{{{\rm{ADC}}}}}_{w}$$

### Agreement analysis between FC-vMRE and MRE

**Fig. 4:**
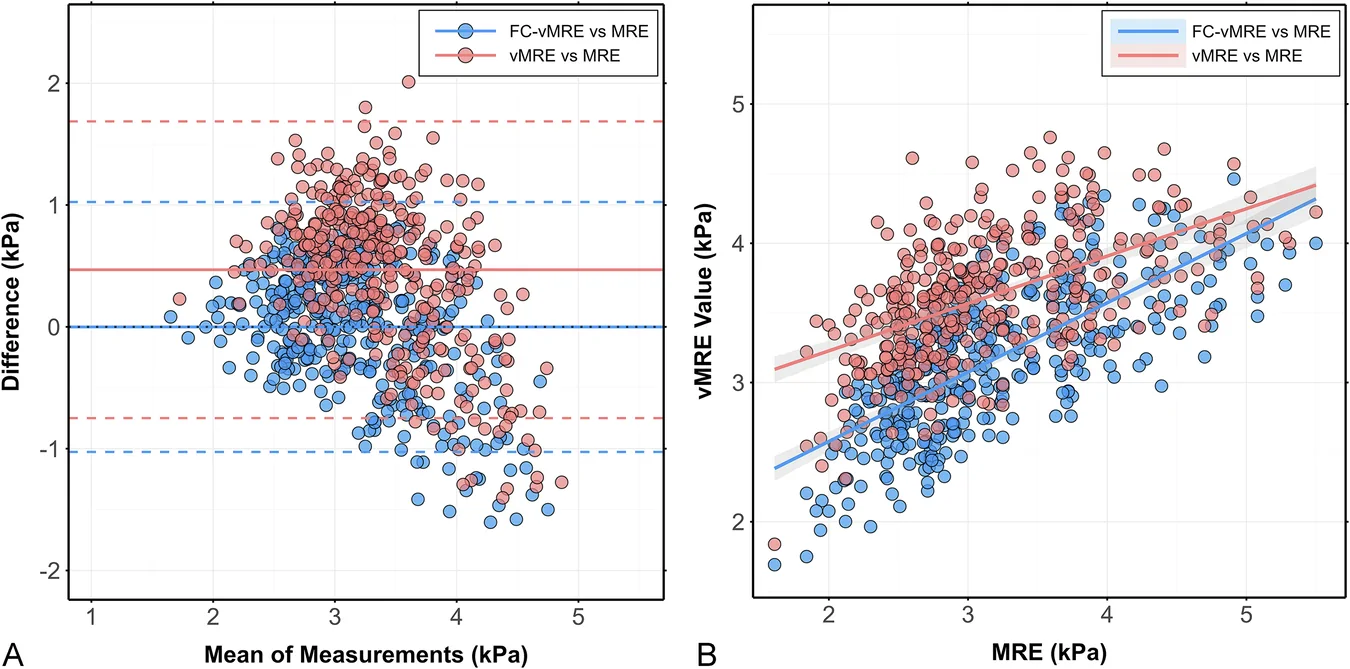
Comparative analysis of liver stiffness measurements: vMRE vs FC-vMRE performance against MRE. **A** Bland–Altman agreement analysis. Bland–Altman plots evaluate the agreement between fat-corrected and uncorrected vMRE methods compared to traditional MRE measurements. **B** Correlation analysis. Scatter plots illustrate the correlations between traditional MRE and both virtual MRE approaches

MRE showed a median liver stiffness of 2.96 kPa (range: 1.61–5.50 kPa). FC-vMRE values exhibited close concordance with MRE measurements (median 3.12 kPa, range 1.69–4.46 kPa), while uncorrected vMRE systematically overestimated liver stiffness (median 3.62 kPa, range 1.84–4.76 kPa). Correlation analysis revealed a significant positive association between FC-vMRE and traditional MRE (r = 0.706, 95% CI [0.65, 0.754], *p* < 0.001), which was significantly stronger than the correlation observed for uncorrected vMRE (r = 0.546, 95% CI [0.47, 0.615]). Bland–Altman analysis further confirmed superior agreement between FC-vMRE and MRE, with a mean bias of −0.001 kPa (95% limits of agreement: −1.027 to 1.025 kPa), significantly lower than the bias observed for uncorrected vMRE (0.469 kPa; 95% limits of agreement: −0.749 to 1.686 kPa). ICC analysis showed good agreement between FC-vMRE and MRE (ICC = 0.666, 95% CI [0.604, 0.719], *p* < 0.001), which was significantly superior to the poor agreement observed for uncorrected vMRE (ICC = 0.381, 95% CI [0.084, 0.579], *p* = 0.006). Comprehensive distribution patterns and comparative analyses of liver stiffness measurements across all three modalities are presented in Fig. 4.

### Diagnostic performance for hepatic fibrosis staging: histopathologic validation

**Fig. 5:**
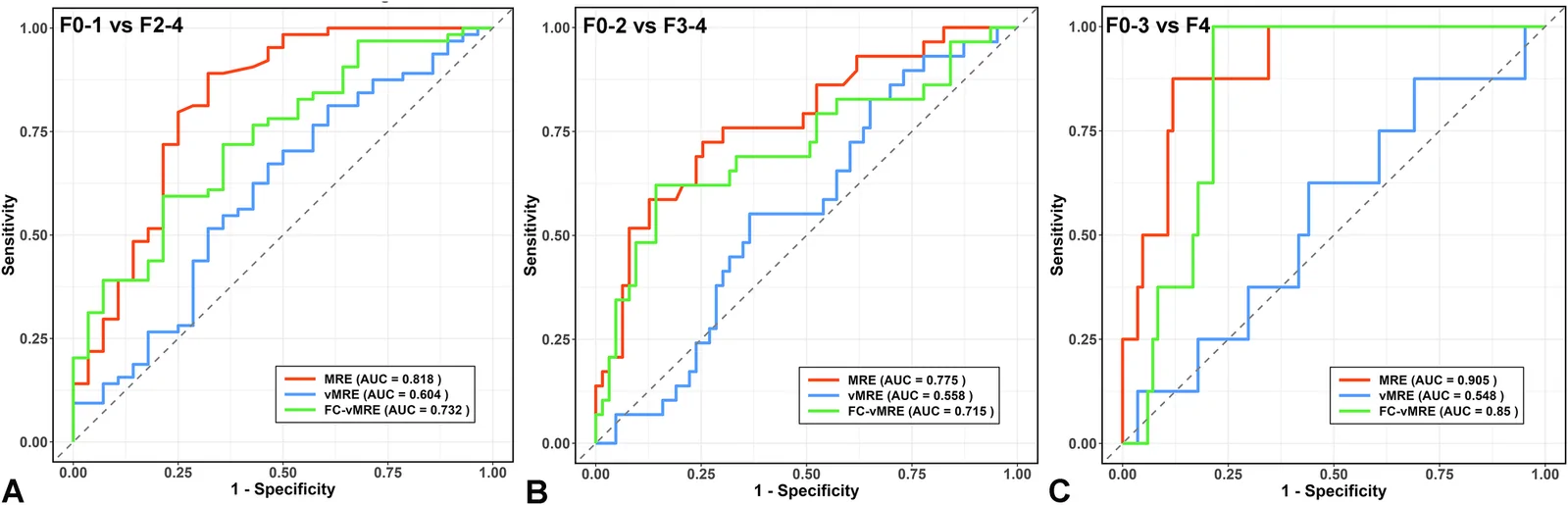
Receiver operating characteristic (ROC) curves comparing diagnostic performance among magnetic resonance elastography (MRE), virtual magnetic resonance elastography (vMRE) and FC-vMRE for liver fibrosis staging in the Validation cohort. **A** Differentiation between early fibrosis (F0-1) and significant fibrosis (F2-4); **B** Distinction between moderate fibrosis (F0-2) and advanced fibrosis (F3-4); **C** Identification of cirrhosis (F4) versus non-cirrhotic stages (F0-3)

**Fig. 6:**
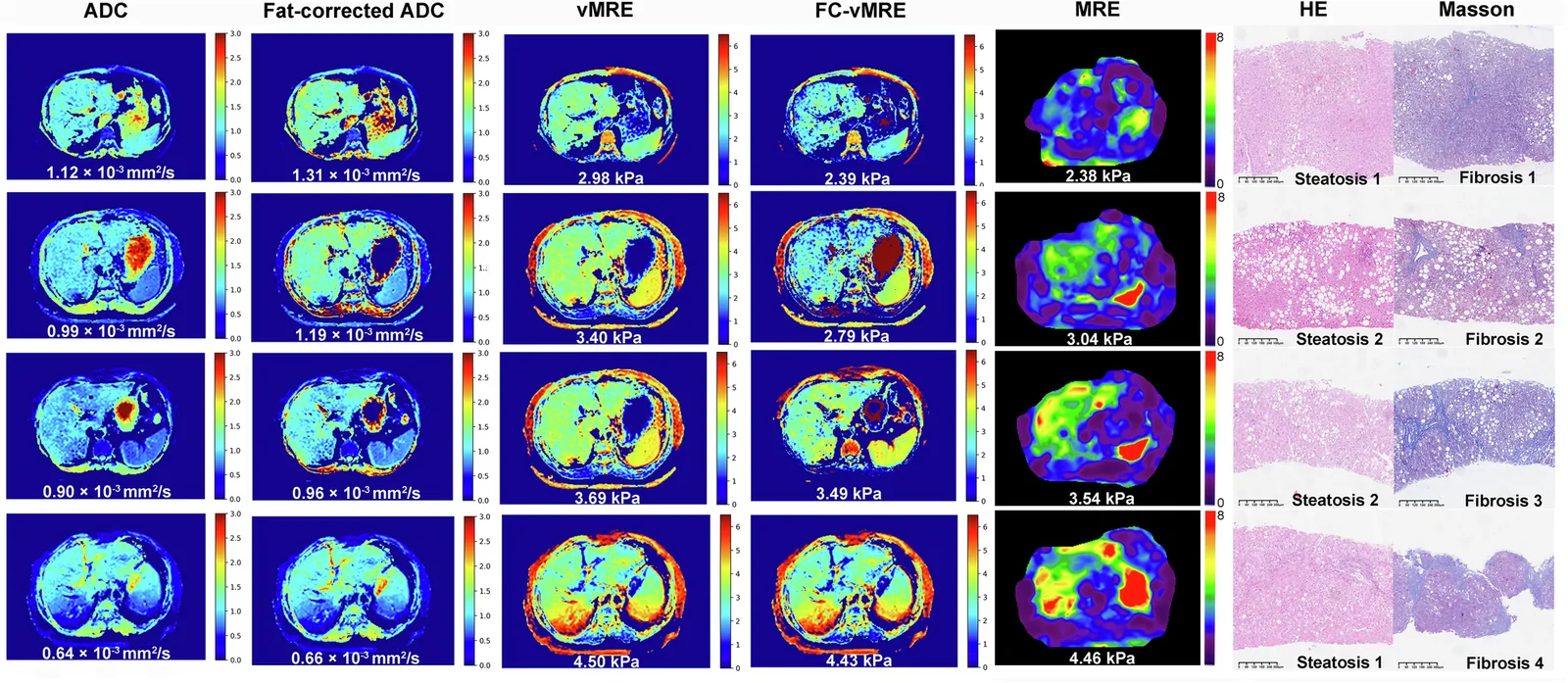
Comparative analysis of vMRE, FC-vMRE and MRE across fibrosis stages in MASLD patients from the validation cohort. Representative cases demonstrate progressive fibrosis severity from top to bottom: F1 (36-year-old male: vMRE 2.98 kPa, FC-vMRE 2.39 kPa, MRE 2.38 kPa), F2 (39-year-old female: vMRE 3.40 kPa, FC-vMRE 2.79 kPa, MRE 3.04 kPa), F3 (63-year-old male: vMRE 3.69 kPa, FC-vMRE 3.49 kPa, MRE 3.54 kPa), and F4 (67-year-old female: vMRE 4.50 kPa, FC-vMRE 4.43 kPa, MRE 4.46 kPa). From left to right: ADC maps, fat-corrected ADC maps, FC-vMRE maps, MRE maps, HE staining (× 6), and Masson’s trichrome staining (× 6)

For detection of significant fibrosis (≥ F2), MRE achieved an AUC of 0.848 (95% CI: 0.757–0.939) with cross-validated performance of 3.09 ± 0.05 kPa cutoff, 87.3 ± 12.5% sensitivity, 70.0 ± 18.9% specificity, and 82.1 ± 10.4% accuracy. FC-vMRE demonstrated comparable performance with an AUC of 0.761 (95% CI: 0.661–0.860), cutoff of 3.42 ± 0.10 kPa, sensitivity of 64.8 ± 17.3%, specificity of 63.3 ± 33.1%, and accuracy of 64.5 ± 9.9%, as illustrated in Fig. 5A. The difference between MRE and FC-vMRE was not statistically significant (DeLong test *p* = 0.053), while MRE significantly outperformed vMRE (*p* = 0.001). FC-vMRE also showed superior performance compared to vMRE (*p* = 0.038). For advanced fibrosis (≥ F3), MRE maintained excellent diagnostic accuracy with an AUC of 0.818 (95% CI: 0.729–0.906) and cross-validated metrics of 3.99 ± 0.13 kPa cutoff, 63.3 ± 18.5% sensitivity, 77.4 ± 12.1% specificity, and 72.5 ± 9.3% accuracy. FC-vMRE achieved an AUC of 0.756 (95% CI: 0.650–0.862) with cutoff of 3.73 ± 0.02 kPa, sensitivity of 62.5 ± 26.7%, specificity of 83.6 ± 14.2%, and accuracy of 76.4 ± 12.0%. The performance difference between MRE and FC-vMRE approached but did not reach statistical significance (*p* = 0.066, Fig. 5B). Both methods significantly outperformed vMRE (AUC = 0.612, 95% CI: 0.499–0.724; *p* < 0.001 and *p* = 0.024, respectively). For cirrhosis detection (F4), the strongest diagnostic performance was observed with MRE, achieving an AUC of 0.914 (95% CI: 0.849–0.978) and cross-validated metrics of 4.28 ± 0.04 kPa cutoff, 80.0 ± 42.2% sensitivity, 85.6 ± 9.1% specificity, and 85.4 ± 8.5% accuracy. FC-vMRE demonstrated robust performance with an AUC of 0.838 (95% CI: 0.761–0.914), cutoff of 3.77 ± 0.01 kPa, sensitivity of 85.0 ± 33.7%, specificity of 77.5 ± 14.8%, and accuracy of 78.3 ± 13.7% (Fig. 5C). The difference between MRE and FC-vMRE was not statistically significant (*p* = 0.065). Both MRE and FC-vMRE significantly outperformed vMRE (AUC = 0.570, 95% CI: 0.392–0.747; *p* < 0.001 and *p* = 0.008, respectively). Detailed cross-validation diagnostic performance metrics for all three modalities across different fibrosis stages are presented in Table 2. Representative MRE, FC-vMRE, and corresponding histopathological images from MASLD patients with varying degrees of liver fibrosis are shown in Fig. 6.

**Table 2:** Cross-validation diagnostic performance of MRE, vMRE, and FC-vMRE for liver fibrosis assessment

	AUC (95% CI)	Cutoff (kPa)	Sensitivity (%)	Specificity (%)	PPV (%)	NPV (%)	Accuracy (%)
F0-1 vs F2-4							
MRE	0.848 (0.757–0.939)	3.09	87.3 ± 12.5	70.0 ± 18.9	87.4 ± 7.2	75.8 ± 22.4	82.1 ± 10.4
vMRE	0.635 (0.514–0.756)	3.39	65.2 ± 18.2	40.0 ± 21.1	71.9 ± 9.0	31.5 ± 15.0	57.8 ± 11.5
FC-vMRE	0.761 (0.661–0.860)	3.42	64.8 ± 17.3	63.3 ± 33.1	83.0 ± 12.1	40.7 ± 16.6	64.5 ± 9.9
F0-2 vs F3-4							
MRE	0.818 (0.729–0.906)	3.99	63.3 ± 18.5	77.4 ± 12.1	61.7 ± 15.8	80.3 ± 9.0	72.5 ± 9.3
vMRE	0.612 (0.499–0.724)	3.39	64.2 ± 25.2	36.4 ± 27.7	36.8 ± 12.4	62.3 ± 27.4	45.9 ± 17.4
FC-vMRE	0.756 (0.650–0.862)	3.73	62.5 ± 26.7	83.6 ± 14.2	69.7 ± 24.1	82.3 ± 12.1	76.4 ± 12.0
F0-3 vs F4							
MRE	0.914 (0.849–0.978)	4.28	80.0 ± 42.2	85.6 ± 9.1	44.2 ± 30.9	97.8 ± 4.7	85.4 ± 8.5
vMRE	0.570 (0.392–0.747)	3.78	35.0 ± 47.4	71.1 ± 25.8	13.0 ± 15.3	88.6 ± 8.9	65.6 ± 20.3
FC-vMRE	0.838 (0.761–0.914)	3.77	85.0 ± 33.7	77.5 ± 14.8	42.5 ± 26.8	97.3 ± 5.8	78.3 ± 13.7

*AUC* area under the curve, *CI* confidence interval, *PPV* positive predictive value, *NPV* negative predictive value, *MRE* magnetic resonance elastography, *vMRE* virtual magnetic resonance elastography, *FC-vMRE* fat-corrected virtual magnetic resonance elastography, *F* fibrosis

## Discussion

This study investigates the potential clinical utility of FC-vMRE derived from DWI as a hardware-free alternative in assessing liver fibrosis in MASLD patients. The incorporation of fat-correction algorithms using PDFF appears to address critical limitations of conventional DWI in MASLD populations where hepatic steatosis confounds ADC measurements. Our findings show that fat-corrected ADC_200-1200_ achieved the strongest correlation with MRE values, suggesting that fat-corrected DWI-based metrics may serve as promising surrogates for liver stiffness measurements. FC-vMRE demonstrated superior agreement with MRE compared to uncorrected vMRE and showed encouraging diagnostic performance for fibrosis staging, positioning FC-vMRE as a promising clinically implementable alternative to MRE.

Our results are consistent with previous studies highlighting the diagnostic potential of DWI in liver disease [14], while extending this to MASLD, characterized by a unique tripartite pathology of steatosis, inflammation, and fibrosis. Optimal *b*-values appear particularly important for MASLD assessment. Our findings suggest that the ADC_200-1200_ offers a strong correlation with MRE for MASLD assessment, differing from previous studies on chronic liver fibrosis favoring 200–1500 [15]. This discrepancy may be attributed to several factors: First, our study focused specifically on MASLD, which encompasses not only fibrosis, steatosis and inflammation, whereas studies like Kromrey et al primarily targeted liver fibrosis. These pathological differences may necessitate different optimal *b*-value combinations. Second, tripartite pathology of MASLD may require different DWI parameters to capture its complex features [16]. Our lower maximum *b*-value (1200 vs 1500) performing well might reflect its ability to detect multifaceted pathological changes in MASLD. Third, our FC-vMRE model underscores that MASLD-specific protocols must account for steatosis-induced ADC alterations [10]. This may explain why a lower maximum *b*-value (1200) performs well, as it might be more sensitive to unique diffusion properties in fatty liver tissue. These findings highlight the importance of tailoring DWI protocols to specific liver diseases, providing insights for optimizing DWI in MASLD assessment.

FC-vMRE demonstrated superior diagnostic performance compared to uncorrected vMRE for liver fibrosis staging and showed encouraging performance compared to MRE across all stages, with no statistically significant differences in the biopsy cohort [17]. The moderate ICC agreement and borderline *p*-values suggest that while FC-vMRE shows promise, its performance should be interpreted with appropriate caution. The observed correlation and agreement metrics between FC-vMRE and MRE, along with minimal measurement bias in the Bland–Altman assessment, support the potential reliability of the fat-corrected approach. This suggests that FC-vMRE could serve as a promising MRE alternative for assessing liver fibrosis in MASLD patients. Our findings contrast with a previous prospective study where FC-vMRE did not predict liver fibrosis in suspected NAFLD patients [18]. This discrepancy highlights that effective fat correction requires integration with MASLD-optimized *b*-values (200/1200 in our study), rather than isolated application. The significantly higher coefficient of determination and lower RMSE of FC-vMRE compared to uncorrected vMRE demonstrate the substantial improvement achieved through fat correction. DWI-derived stiffness measurements in vMRE appear to capture the mechanical properties of fibrotic liver tissue, despite utilizing fundamentally different physical principles than direct mechanical wave propagation in MRE [19]. The cutoff values for FC-vMRE established in our study, while not identical, showed general alignment with existing MRE literature, supporting their potential as useful diagnostic thresholds [14]. These cutoffs maintained balanced sensitivity and specificity across clinically crucial fibrosis stages.

For advanced fibrosis and cirrhosis detection, FC-vMRE demonstrated encouraging diagnostic performance, maintaining reasonable sensitivity and specificity. The comparable diagnostic accuracy between FC-vMRE and MRE further reinforces FC-vMRE as a reliable alternative to MRE. Notably, FC-vMRE showed consistently superior performance compared to uncorrected vMRE across all fibrosis stages. The dramatic improvement in diagnostic accuracy from vMRE to FC-vMRE underscores the critical importance of accounting for hepatic steatosis. The superior diagnostic performance of FC-vMRE over uncorrected vMRE may relate to its correction for steatosis-induced confounding effects on water diffusion characteristics [20, 21]. Unlike MRE, which measures macroscopic tissue displacement, FC-vMRE foundation in fat-corrected ADC values makes it potentially more sensitive to microstructural changes in hepatocyte integrity and extracellular matrix composition [22]. This property might enable FC-vMRE to better assess fibrosis while minimizing steatosis interference, particularly during early stages where both MRE and uncorrected vMRE face limitations. The slightly lower sensitivity of FC-vMRE compared to MRE suggests that while promising, FC-vMRE may have limitations in detecting early fibrosis stages compared to direct measurement of tissue mechanical properties of MRE.

Optimizing *b*-values for DWI acquisition in MASLD patients represents a significant study contribution. Previous vMRE studies may not have accounted for the unique pathological triad of MASLD [8, 14, 18]. Our findings suggest *b*-values of 200–1200 s/mm² provide optimal correlation with liver stiffness in MASLD patients. This optimization may improve vMRE accuracy in this population and guide future DWI protocol development for metabolic liver diseases. Leveraging existing DWI data for liver stiffness estimation, vMRE could potentially increase liver fibrosis assessment accessibility. vMRE offers several advantages over MRE, including reduced examination times, enhanced accessibility, and lower costs. This is particularly relevant where MRE hardware is unavailable or contraindicated. The ability to perform liver evaluation without additional hardware could also improve the assessment of liver heterogeneity, which is often a challenge in MASLD due to the patchy nature of disease progression. Moreover, the encouraging performance of FC-vMRE to MRE in fibrosis staging and MASH detection suggests that it could serve as a useful tool for longitudinal monitoring of MASLD patients. This could be particularly useful in assessing treatment response and disease progression over time, potentially reducing the need for repeated liver biopsies.

Several limitations warrant consideration. First, the single-center design and specific MRI system may limit generalizability across different institutions and imaging platforms. Second, the relatively small validation cohort with biopsy confirmation may not fully represent the diverse MASLD population spectrum. Third, the fat-correction algorithm relies on predetermined tissue parameters derived from prior literature, which may not accurately reflect individual patient variations or different stages of liver disease. Future multicenter studies with larger cohorts and different MRI platforms are needed to validate these findings.

In conclusion, FC-vMRE represents a promising noninvasive technique for assessing liver fibrosis and MASH in MASLD patients. With performance showing encouraging concordance with MRE while offering practical advantages of no additional hardware requirements and reduced costs, FC-vMRE presents a potentially valuable option for widespread clinical implementation, particularly where MRE is unavailable.

## Supplementary information

ELECTRONIC SUPPLEMENTARY MATERIAL

## Data Availability

The dataset used or analyzed during the current study is available from the corresponding author on reasonable request.

## Abbreviations

ADC: Apparent diffusion coefficient
ADC_*w*_: Fat-corrected apparent diffusion coefficient
DWI: Diffusion-weighted imaging
FC-vMRE: Fat-corrected vMRE
MASH: Metabolic dysfunction-associated steatohepatitis
MASLD: Metabolic dysfunction-associated steatotic liver disease
MRE: Magnetic resonance elastography
MRI: Magnetic resonance imaging
PDFF: Proton density fat fraction
vMRE: Virtual magnetic resonance elastography
