# Correction: Development and validation of fat-corrected virtual MR elastography to assess fibrosis stage in metabolic dysfunction-associated steatotic liver disease

**DOI:** 10.1186/s13244-026-02369-3

**Published:** 2026-09-10

**Authors:** Jie Yuan, Xinxin Xu, Fuhua Yan, Zhiwei Qin, Huamei Yan, Wenjie Yang, Huimin Lin

**Affiliations:** 1 Department of Radiology, Ruijin Hospital, Shanghai Jiao Tong University School of Medicine, Shanghai, China; 2 Department of Radiology, Shuguang Hospital Affiliated to Shanghai University of Traditional Chinese Medicine, Shanghai, China; 3 College of Health Science and Technology, Shanghai Jiao Tong University School of Medicine, Shanghai, China; 4 MR Research Collaboration, Shanghai United Imaging Healthcare Co., Ltd., Shanghai, China; 5 Clinical Research Center, Shuguang Hospital Affiliated to Shanghai University of Traditional Chinese Medicine, Shanghai, China

**Correction to: Insights into Imaging**

https://doi.org/10.1186/s13244-026-02287-4, published online 18 April 2026

**The original paper contained a copying error within Table 2. The corrected version of Table 2 is below.**

**Table 2:** Cross-validation diagnostic performance of MRE, vMRE, and FC-vMRE for liver fibrosis assessment

	AUC (95% CI)	Cutoff (kPa)	Sensitivity (%)	Specificity (%)	PPV (%)	NPV (%)	Accuracy (%)
F0-1 vs F2-4							
MRE	0.848 (0.757–0.939)	3.09	87.3 ± 12.5	70.0 ± 18.9	87.4 ± 7.2	75.8 ± 22.4	82.1 ± 10.4
vMRE	0.635 (0.514–0.756)	3.39	65.2 ± 18.2	40.0 ± 21.1	71.9 ± 9.0	31.5 ± 15.0	57.8 ± 11.5
FC-vMRE	0.761 (0.661–0.860)	3.42	64.8 ± 17.3	63.3 ± 33.1	83.0 ± 12.1	40.7 ± 16.6	64.5 ± 9.9
F0-2 vs F3-4							
MRE	0.818 (0.729–0.906)	3.99	63.3 ± 18.5	77.4 ± 12.1	61.7 ± 15.8	80.3 ± 9.0	72.5 ± 9.3
vMRE	0.612 (0.499–0.724)	3.39	64.2 ± 25.2	36.4 ± 27.7	36.8 ± 12.4	62.3 ± 27.4	45.9 ± 17.4
FC-vMRE	0.756 (0.650–0.862)	3.73	62.5 ± 26.7	83.6 ± 14.2	69.7 ± 24.1	82.3 ± 12.1	76.4 ± 12.0
F0-3 vs F4							
MRE	0.914 (0.849–0.978)	4.28	80.0 ± 42.2	85.6 ± 9.1	44.2 ± 30.9	97.8 ± 4.7	85.4 ± 8.5
vMRE	0.570 (0.392–0.747)	3.78	35.0 ± 47.4	71.1 ± 25.8	13.0 ± 15.3	88.6 ± 8.9	65.6 ± 20.3
FC-vMRE	0.838 (0.761–0.914)	3.77	85.0 ± 33.7	77.5 ± 14.8	42.5 ± 26.8	97.3 ± 5.8	78.3 ± 13.7

*AUC* area under the curve*, CI* confidence interval*,* *PPV* positive predictive value*, NPV* negative predictive value*, MRE* magnetic resonance elastography*, vMRE* virtual magnetic resonance elastography*, FC-vMRE* fat-corrected virtual magnetic resonance elastography*, F* fibrosis

