# Supplementary material for: Development and validation of fat-corrected virtual MR elastography to assess fibrosis stage in metabolic dysfunction-associated steatotic liver disease
Source: Insights Imaging. 2026 Apr 18;17:103. doi: 10.1186/s13244-026-02287-4 (PMC13091820; doi:10.1186/s13244-026-02287-4)
Supplement: Supplementary file 1 — ELECTRONIC SUPPLEMENTARY MATERIAL [file 13244_2026_2287_MOESM1_ESM.pdf]

Development and Validation of Fat-corrected Virtual MR Elastography to Assess Fibrosis Stage in Metabolic Dysfunction-Associated Steatotic Liver Disease

ELECTRONIC SUPPLEMENTARY MATERIAL

Inter-reader Reproducibility Analysis

Inter-reader reproducibility analysis demonstrated excellent agreement across all imaging parameters. For MRE, the ICC was 0.977 (95% CI: 0.963-0.986) with a mean difference of -0.042 kPa and coefficient of variation (CoV) of 5.58%. PDFF measurements showed outstanding reproducibility with an ICC of 0.997 (95% CI: 0.995-0.998), mean difference of 0.021%, and CoV of 4.88%.

All ADC parameters demonstrated good to excellent inter-reader agreement, with ICC values ranging from 0.901 to 0.983. Parameters with lower *b*-values showed ICC values between 0.942 and 0.983, while those incorporating higher *b*-values showed slightly lower but still acceptable ICC values (0.901-0.955). Detailed inter-reader reproducibility metrics for all parameters are presented in Table S1.

Table S1. Inter-reader Reproducibility Results.

Parameter	ICC (95% CI)	Mean Difference	SD Difference	CoV (%)
MRE (kPa)	0.977 (0.963-0.986)	-0.042 kPa	0.184	5.58
PDFF (%)	0.997 (0.995-0.998)	0.021%	0.660	4.88
ADC Parameters ($\times 10^{-3}$ mm^2/s)				
ADC_{0-800}	0.981 (0.970-0.988)	0.005	0.057	3.86
ADC_{0-1000}	0.983 (0.973-0.989)	-0.002	0.040	2.96
ADC_{0-1200}	0.983 (0.974-0.990)	-0.004	0.036	2.89
ADC_{0-1500}	0.981 (0.969-0.988)	-0.003	0.031	2.81
ADC_{50-800}	0.967 (0.948-0.979)	0.008	0.058	5.17
$ADC_{50-1000}$	0.973 (0.957-0.983)	0.000	0.040	3.79
$ADC_{50-1200}$	0.972 (0.955-0.982)	-0.003	0.036	3.62
$ADC_{50-1500}$	0.967 (0.947-0.979)	-0.002	0.034	3.70
$ADC_{100-800}$	0.948 (0.918-0.967)	0.009	0.071	6.52
$ADC_{100-1000}$	0.964 (0.944-0.978)	0.000	0.050	4.83
$ADC_{100-1200}$	0.961 (0.939-0.976)	-0.003	0.043	4.47
$ADC_{100-1500}$	0.966 (0.947-0.979)	-0.002	0.036	4.04
$ADC_{150-800}$	0.942 (0.908-0.964)	0.021	0.083	7.85
$ADC_{150-1000}$	0.954 (0.927-0.971)	0.009	0.059	5.87
$ADC_{150-1200}$	0.959 (0.936-0.974)	0.005	0.049	5.18
$ADC_{150-1500}$	0.966 (0.946-0.979)	0.003	0.039	4.49
$ADC_{200-800}$	0.901 (0.847-0.937)	0.016	0.112	10.73
$ADC_{200-1000}$	0.912 (0.863-0.944)	0.005	0.078	7.91
$ADC_{200-1200}$*	0.929 (0.890-0.955)	0.001	0.059	6.33
$ADC_{200-1500}$	0.955 (0.929-0.972)	0.000	0.042	4.81

Calibration Analysis

For ≥F2 detection, FC-vMRE showed excellent calibration with a slope of 1.038, intercept of -0.026, and Hosmer-Lemeshow $\chi^2 = 8.33$ ($p = 0.402$). Similarly, for ≥F3 detection, calibration metrics showed slope = 1.083, intercept = -0.027, and $\chi^2 = 13.45$ ($p = 0.097$). For cirrhosis, FC-vMRE maintained good calibration (slope = 0.959, intercept = 0.005, $\chi^2 = 10.93$, $p = 0.205$). In comparison, MRE demonstrated comparable calibration performance (≥F2: slope = 1.051, intercept = -0.035, $p = 0.100$; ≥F3: slope = 0.972, intercept = 0.009, $p = 0.493$; F4: slope = 1.029, intercept = -0.003, $p = 0.952$), while vMRE showed less optimal calibration, particularly for cirrhosis detection (slope = 1.318, intercept = -0.036, $p = 0.959$). All calibration plots are presented in Fig. S1.

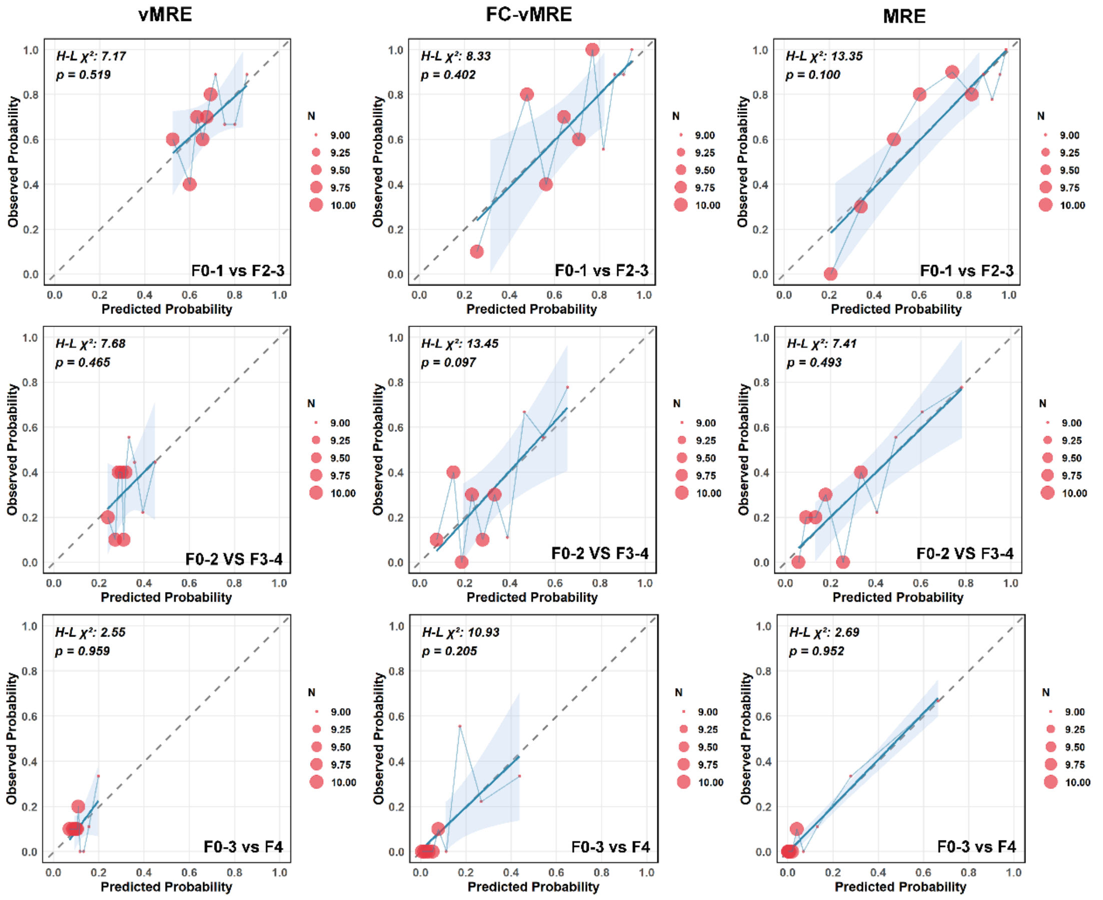

Fig S1. Calibration performance of FC-vMRE, vMRE, and MRE for fibrosis staging in the validation cohort.
